# An Interpretable Classification Framework for Information Extraction from Online Healthcare Forums

**DOI:** 10.1155/2017/2460174

**Published:** 2017-08-03

**Authors:** Jun Gao, Ninghao Liu, Mark Lawley, Xia Hu

**Affiliations:** ^1^Department of Computer Science and Engineering, Texas A&M University, College Station, TX, USA; ^2^Department of Industrial and Systems Engineering, Texas A&M University, College Station, TX, USA; ^3^Center for Remote Health Technologies and Systems, Texas A&M Engineering Experiment Station, College Station, TX, USA

## Abstract

Online healthcare forums (OHFs) have become increasingly popular for patients to share their health-related experiences. The healthcare-related texts posted in OHFs could help doctors and patients better understand specific diseases and the situations of other patients. To extract the meaning of a post, a commonly used way is to classify the sentences into several predefined categories of different semantics. However, the unstructured form of online posts brings challenges to existing classification algorithms. In addition, though many sophisticated classification models such as deep neural networks may have good predictive power, it is hard to interpret the models and the prediction results, which is, however, critical in healthcare applications. To tackle the challenges above, we propose an effective and interpretable OHF post classification framework. Specifically, we classify sentences into three classes: medication, symptom, and background. Each sentence is projected into an interpretable feature space consisting of labeled sequential patterns, UMLS semantic types, and other heuristic features. A forest-based model is developed for categorizing OHF posts. An interpretation method is also developed, where the decision rules can be explicitly extracted to gain an insight of useful information in texts. Experimental results on real-world OHF data demonstrate the effectiveness of our proposed computational framework.

## 1. Introduction

The past few years have witnessed the increasing popularity of online health forums (OHFs), such as WebMD Discussions and Patient, as communication platforms among patients. According to a survey by PwC in 2012, 54% of 1060 participants are comfortable with their doctors getting information related to their health conditions from online physician communities [1]. OHFs can be used for patients to ask for suggestions and share experiences. The abundant user-generated content related to healthcare on the OHFs could provide insightful information to the other patients, medical doctors, and decision makers to promote the understanding about diseases and the health conditions of patients.

**Figure 1:**
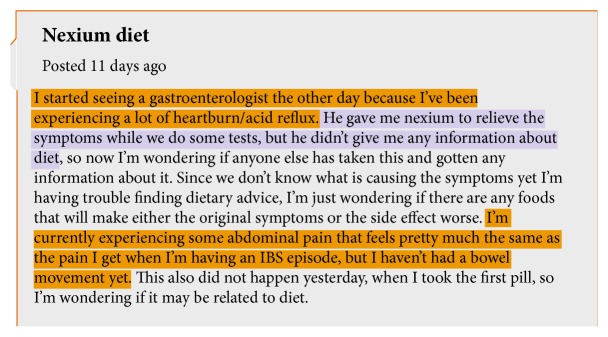
An example of an online health forum post.

To extract insightful information from OHF posts, a commonly adopted strategy is to split posts into sentences and classify each sentence into different categories according to their semantical meanings [2, 3]. For example, Figure 1() shows a post from an OHF called patient.info (https://patient.info/forums). We highlight the sentences about symptoms in orange, and the one about medication in violet. The former ones provide the information about the user's symptoms, reflected by the terms “heartburn,” “acid reflux,” “abdominal pain,” and “IBS.” The latter one tells the user's medication treatment, where the term “nexium” presents the medication for the disease. These pieces of information can help other users to gain a more comprehensive understanding of the disease.

However, it is a challenging task to effectively analyze the expressions in online health forums. First, the user-generated content in OHFs is usually unstructured and contains background information that is relatively less important to analyze [3]. The irregularity and noises in data impede us from directly applying existing classification models to analyze posts automatically. A more sophisticated classification framework is needed for processing unstructured data in OHFs, in order to extract useful patterns (e.g., terms, text sequences) for accurate categorization. Second, when categorizing post sentences into different classes, it is difficult to make the tradeoff between classification accuracy and interpretability [4, 5]. In health-related tasks, besides desirable classification performance, human-understandable explanations for classification results are also crucial, because patients or doctors will not take the risk to trust the predictions they do not understand. Complex models (e.g., deep neural networks, SVM) are accurate in classification, but they do not directly provide the reasons for individual classification results. Simple models such as linear classifiers and decision trees can provide interpretations along with classification outcomes, but usually they cannot achieve comparable performances as complex models.

In this paper, we propose an effective framework for analyzing OHF posts. We propose to develop a random forest model to classify the sentences into three categories, that is, medication, symptom, and background, in order to get an accurate understanding of the role of each sentence in the overall expression of the health situation. Besides, human-understandable interpretations for classification results are generated for the forest model. To enable interpretation, the features involved in the classification task are designed in a human-understandable manner. Moreover, the contribution of features to a classification instance can be explicitly measured by the decision rules constructed during training process [6–8]. Specifically, we represent healthcare-related sentences with various semantic features such as labeled sequential patterns (LSPs), UMLS semantic type features [3], and sentence-based and heuristic features. LSPs represent the frequent tag-based patterns in texts. UMLS features indicate the existence terminologies defined by domain experts. In this way, each unstructured sentence is mapped to the feature space which facilitates further analysis. Also, word-based and heuristic information can also be used to enhance the classification performance. The contributions of this paper are summarized as follows:

- We propose a forest-based framework to deal with the healthcare-related text classification problem. Labeled sequential pattern features are involved in characterizing the unstructured healthcare-related texts from both syntactic and semantic levels.
- We develop a method for constructing decision rules integrated from decision trees in forest-based models to achieve model interpretability.
- The effectiveness and interpretability of our framework are demonstrated through experiments on a real OHF dataset, where we analyze the interpretations provided by our framework in detail.

## 2. Framework Overview

**Figure 2:**
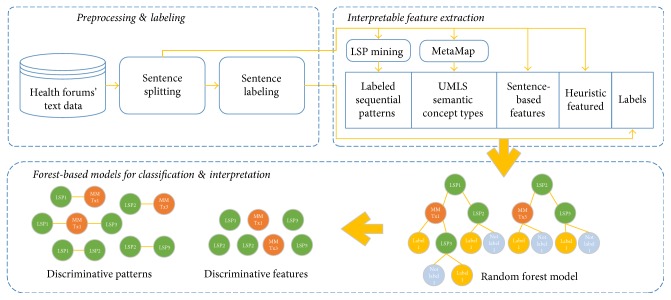
An overview of the interpretable classification framework.

In this section, we will briefly introduce each module of our proposed framework (Figure 2) including data preprocessing, interpretable feature extraction, and forest-based models for classification and interpretation. We categorize each sentence of posts into one of the three categories: *medication*, *symptom*, and *background.* The definition of each category is given as follows.

- *Medication:* If a sentence contains information relevant to curing diseases, treating any medical conditions, relieving any symptoms of diseases, or preventing any diseases, then we assign the sentence to the *medication* category.
- *Symptom:* If a sentence contains any contents relevant to departures from normal functioning or feelings of individuals, which may express the phenomenon affected by diseases, we assign the sentence to the *symptom* category.
- *Background:* If a sentence cannot be classified to the medication or symptom category, then we assign the sentence to *background* category.

Given a sentence “I am taking 90 units Lantus twice a day” for classification, for example, we will first convert it into an instance in a feature space through preprocessing to identify the number term “90,” the drug term “Lantus,” the frequency term “twice a day,” the context of each term, and so forth. Then, we will use the forest-based model to classify the sentence, along with the explanations based on the discriminative features identified by the model.

### 2.1. Module 1: Preprocessing and Labeling

In this module, we split the collected online health community posts into sentences and manually assign each sentence one label from the classes {*medication*, *symptom*, *background*}. Formally, let ℍ be the healthcare-related natural language space and *𝕃* = {*medication*, *symptom*, and *background*} be the target label space. Suppose a collection of *N* labeled sentences

(1) $$\begin{array}{c} S=\left\{\left(s_{i},l_{i}\right)\;|\;1\leq i\leq N,s_{i}\in ℍ,l_{i}\in L\right\} \end{array}$$

are available for model training and testing; *s*_*i*_ represents the original text of the *i*th sentence and *l*_*i*_ represents the label of the *i*th sentence. In other words, each sentence is labeled as *medication*, *symptom*, or *background*.

### 2.2. Module 2: Interpretable Feature Extraction

In this module, we propose the feature extraction method *f* : ℍ  →  ℝ^*D*^ to convert healthcare-related sentences into instances in a *D*-dimensional numerical space, where *D* is the number of features used to represent each sentence. In this way, we can represent each unstructured sentence with a numerical vector, which facilitates model training and testing. After that, the overall dataset is transformed to

(2) $$\begin{array}{c} X=f\left(S\right)=\left\{\left(x_{i},l_{i}\right)\;|\;1\leq i\leq N\right\}, \end{array}$$

where *𝒮* is the original labeled sentence dataset and *N* is the number of sentences, while **x**_*i*_ = (*x*_1_, *x*_2_,…, *x*_*D*_) is the resultant numerical instance represented by *D* features. These features are also intuitive and insightful to help people better understand the sentences. We will discuss this module in detail in Section 3.

### 2.3. Module 3: Forest-Based Models for Classification and Interpretation

In this module, the task is to train a model *F* : ℝ^*D*^  →  *𝕃* that can classify an instance into a class from {*medication*, *symptom*, or *background*} and interpret the sentences belonging to class *medication* and *symptom*. We mainly introduce building forest-based models to classify and interpret the instances: (1) random forests [9] are grown on the numerical instances obtained from the feature engineering module, which can be interpreted by the features of higher importance according to some criterion, for example, Gini impurity. (2) DPClass [8] is a method based on random forest models to extract discriminative combinations of decision rules in the forest, which can be implemented by using forward selection to choose the top combinations. This module will be discussed in detail in Section 4.

## 3. Extracting Interpretable Features

Interpretable features play an essential role in enabling users to understand prediction results. In this section, we discuss how to convert health-related sentences into instances in numerical feature space composed of labeled sequential patterns, UMLS semantic type features, sentence-based features, and heuristic features. The method of extracting labeled sequential patterns is introduced in detail.

### 3.1. Labeled Sequential Patterns

In sentence classification, if we simply use bag of words to represent each sentence, the overall data matrix will be huge and sparse, because there are a large number of terms, and many terms only occur in few sentences about some specific diseases. It is undesirable to use these raw terms to explain their correlations with sentence category as interpretations for classification results. The reason is that the raw terms do not explicitly specify the semantics of words, or contain the structural information of sentences. Therefore, we propose to use higher-level features to represent a sentence rather than words. We will rely on these higher-level features to interpret the sentences classification results.

#### 3.1.1. Labeled Sequence Mapping

We first extract *labeled sequences* as preliminary representations of sentences [10]. A labeled sequence is in the form *sequence* → *label*, where *sequence* is a sequence of tags and *label* is the class label. To convert a sentence into a sequence, we use the tags in Table 1 to replace the words in the sentence. Words with similar semantics are mapped to the same tag. For example, the medication sentence “I am taking 90 units Lantus twice a day” can be converted into tag-word pairs ((*PRP*, “*I*”), (*VBP*, “*am*”), (*VBG*, “*taking*”), (*CD*, “*90*”), (*NNS*, “*units*”), (*DRUG*, “*Lantus*”), (*FREQ*, “*twice a day*”)) and the entire sentence is represented as a labeled sequence: (*PRP*, *VBP*, *VBG*, *CD*, *NNS*, *DRUG*, *FREQ*) → *medication.*

Given a training set of labeled sentences *𝒮* = {(*s*_1_, *l*_1_),…, (*s*_*n*_, *l*_*n*_)}, we convert each pair into a labeled sequence *p*_*i*_  →  *l*_*i*_ by applying the method mentioned above so that we can obtain the database *𝒟* of labeled sequences. Our next goal is to mine the frequent patterns in the labeled sequences from *𝒟* and adopt these frequent patterns as features to capture the characteristics of the healthcare-related sentences. This task can be divided into two steps: (1) frequent sequential pattern mining and (2) building frequent labeled sequential patterns.

#### 3.1.2. Frequent Sequential Pattern Mining

We now focus on mining the frequent sequential patterns from database *𝒟*. Before that, we first define sequential pattern as follows.

**Definition 1 .**

A *sequential pattern* is a sequence of tags which is a subsequence of one or more *sequences* in the database. The adjacent tags are not necessarily adjacent in the original sequences, but their distance should be not greater than a threshold in the original sequences, which is set as 5 in experiments [10].

For example, given two labeled sequences (*a*, *b*, *c*, *d*, *e*, *f*)  →  *l*_1_ and (*a*, *c*, *d*, *e*, *g*, *h*)  →  *l*_2_ in the database *𝒟*, (*a*, *c*, *e*) can be considered as a sequential pattern of both *sequence*s. Note that a *sequence* is different from a labeled sequence. The former only consists of the sequence of tags, while the latter includes the mapping from sequence to the label, that is, *P*_*i*_  →  *l*_*i*_.

**Definition 2 .**

A *frequent sequential pattern* (FSP) is a *sequential pattern p*′ with sup(*p*′) ≥ *μ*, where *μ* is a customized threshold and sup(*p*′) denotes the support of *p*′ in *𝒟*, that is,

(3) $$\begin{array}{c} \sup\left(p^{\prime }\right)=\frac{\left|\left\{p\;|\;p\;contains\;p^{\prime },p\in D\right\}\right|}{\left|D\right|}, \end{array}$$

where *p* is any *sequence* in the database *𝒟* that contains *p*′. sup(*p*′) represents the percentage of the sequences in the database that contain *p*′, which shows the generality of *p*′ in the database *𝒟*.

There are several algorithms to mine frequent patterns from a database. We select CM-SPAM [11] to obtain FSPs from *𝒟*. The minimum threshold *μ* is customized by users such that the resultant FSPs would be general enough.

#### 3.1.3. Frequent Labeled Sequential Patterns

With FSPs available, the next step is to select a subset of promising FSPs called frequent labeled sequential patterns (FLSPs) which are then used for classification.

Note that we have two classes: *medication* and *symptom*; thus, the FLSPs are different for each class. Formally, an FLSP of label *l* is defined as the FSP with high *confidence* with respect to *l*. Given a specific label *l*, the confidence of a frequent sequential pattern *p*′, denoted by conf (*p*′), is computed as

(4) $$\begin{array}{c} conf\left(p^{\prime }\right)=\frac{\left|\left\{p\;|\;p\;contains\;p^{\prime },p\;\to \;l\in D\right\}\right|}{\left|\left\{p\;|\;p\;contains\;p^{\prime },p\in D\right\}\right|}, \end{array}$$

which is the ratio of *sequences* that contain the FSP *p*′ and are labeled *l* to the *sequences* containing the FSP *p*′. FSPs with high confidence show strong relations to the given label *l*, since a large portion of those frequent sequential patterns are labeled as *l*.

We would also like to set the minimum support threshold to a small percentage in order to include more FSPs. In our experiments, we set the minimum *support* to 5%. Besides, the minimum confidence threshold might also not necessarily be set very large since we would like to obtain more FLSPs by reducing some predictive ability of them in the early stage. In the experiments, we set the minimum *confidence* to 85% [10].  Algorithm 1 shows the entire process of generating FLSPs from text data.

Finally, we obtain a set of FLSPs, which can be used as features to identify the relationship between labels and patterns in sentences [12]. We use each frequent labeled sequential pattern as a feature. For each instance in the training set, if its mapped *sequence* contains a FLSP, we will set the value of the corresponding feature entry to 1; otherwise 0.

### 3.2. UMLS Metathesaurus Semantic Types

In addition to FLSPs, we also use UMLS [13] Metathesaurus semantic types as features. There are 133 UMLS Metathesaurus semantic types in total. By using the third-party software MetaMap (https://mmtx.nlm.nih.gov/) [14], we can map the sentence to these semantic types (https://mmtx.nlm.nih.gov/Docs/SemanticTypes_2013AA.txt). Thus, for each semantic type feature, we set the value to 1 if the sentence contains any phrases related to the semantic type; otherwise, 0.

Generally, for each sentence *s*_*i*_ in *𝒮*, it is converted into **x**_**i**_ which is a representation vector of the sentence in the feature space of FLSPs and the UMLS semantic types. If *s*_*i*_ contains any FLSPs or phrases related to UMLS semantic types, the value of the corresponding feature entry in **x**_**i**_ is set to 1.

### 3.3. Sentence-Based Features

Sentence-based features are capable of representing the sentence in a direct way [3]. In this paper, we use the following sentence-based features to represent sentences.

#### 3.3.1. Word-Based Features

Although word-based features such as bag-of-word representation usually suffer from the curse of dimensionality, we still take them into account to compare the classification performance because of their effectiveness [15]. Unigrams and bigrams can capture those significant and frequent words or phrases related to a specific label. For example, it is likely that a sentence is classified into medication category if the word “prescribe” occurs. Each unigram or bigram corresponds a binary feature to indicate if a sentence contains this feature or not.

#### 3.3.2. Morphological Features

Capitalized words and abbreviations can be good indicators of whether there are any medical terminologies in the sentence, which could be highly related to medication or symptom sentences. We can use two binary features to indicate whether the sentence contains any capitalized words or abbreviations, respectively.

### 3.4. Heuristic Features

In addition to all the features originated from the texts of the sentences, we can also adopt useful side information of posts [3]. Specifically, a sentence written by the thread creator is more likely to be symptom-related compared to the one written by the other users, because thread creators tend to ask for help from other users by posting their own conditions. Besides, the position of the post which a sentence is from can also indicate the category, because the first post written by the thread creators are usually describing the patients' situations, while the latter posts tend to answer the potential questions that arise in the first couple of posts. Thus, two binary features are considered to indicate whether a sentence is written by the thread creator, and the position of the post which the sentence is from, respectively.

In general, we can select different combinations of the features introduced in this section to represent health-related sentences and then build models to predict the categories of sentences with interpretations.

## 4. Interpretable Classification with Forest-Based Models

In this section, we first introduce the classification of health forum sentences using a random forest model and how to interpret the forest model with features of high importance. Second, we introduce how to collect rules from decision trees in the forest to construct a new pattern space [8] and achieve the interpretability by selecting the top patterns.

### 4.1. Classification with Random Forests

A random forest consists of an ensemble of tree-based classifiers and calculates the votes from the trees for the most popular class in classification problems [9]. The growth of the ensemble is determined by the growth of each tree therein. The process of tree growth is introduced as follows [16]:

1. Sample *N*_*T*_ instances at random with replacement from the training set. The samples will then be used to grow the tree model.
2. A subset of *m* features are selected from the total *D* features at random, where *m* ≪ D. The best split on the *m* features will be used to construct the tree nodes such that the Gini impurity for the descendants will be less than that of the parent node, using the method introduced in CART [17]. The value of *m* remains constant during the forest growing process.
3. Each tree grows to the maximum size without pruning.

When growing a tree using the samples from the original training set, about one-third of the instances in the training set are left out of the samples selected at random. This out-of-bag data will be an unbiased estimate of the classification accuracy for the currently growing tree and also can be used to estimate features importance.

### 4.2. Interpretation with Discriminative Features

The classification mechanism of a random forest is explained through a set of decision paths. To interpret random forest models, we propose to quantify the contributions of node features, rank them according to their contributions, and find out the most discriminative ones [7, 18].

For a decision tree in the random forest, its decision function can be formulated as follows:

(5) $$\begin{array}{c} f\left(x\right)=\overset{M}{\underset{m=1}{\sum }}c_{m}I\left(x,R_{m}\right), \end{array}$$

where *M* is the number of leaf nodes in the tree. *c*_*m*_ denotes the criterion score, which is a scalar in regression problems or a vector in classification problems, learned from the training process. **x** is the input sample. *R*_*m*_ is the path from the root to the *m*th leaf node. *I*(·, ·) is an indicator function identifying whether *x* is run through *R*_*m*_. As we are solving a classification problem, *c*_*m*_ and *f*(**x**) should be vectors whose sizes are the number of the classes. The *i*th value in the vector *f*(**x**) represents the criterion score of the instance **x** being classified into the *i*th class, which can be converted to a probability value by normalization. In our problem of classification, an input instance **x** is classified into one class from the classes {*medication*, *symptom*, *background*} according to the maximum probability specified by *f*(**x**) of the decision tree.

From another perspective, we can observe how a feature contributes to the *criterion score* (i.e., Gini impurity or entropy) vector by calculating the score vector difference between the current node and the next node in the path. The final prediction result along a tree path is determined under the cumulative influences of nodes in the path. Therefore, a prediction can be defined as a sum of feature contributions plus a bias:

(6) $$\begin{array}{c} f_{t}\left(x\right)=\overset{D}{\underset{k=1}{\sum }}{FC}_{t,k}\left(x\right)+\beta_{t}, \end{array}$$

where *FC*_*t*,*k*_(**x**) is the feature contribution vector from the *k*th feature in the *t*th tree for an input vector **x**, *D* is the number of features, and *β*_*t*_ is the bias of tree *t*. Both *FC*_*t*,*k*_(**x**) and *β*_*t*_ are criterion score vectors. Our goal is to calculate the feature contributions for an instance **x** classified by a decision tree *t* that has been trained on the training set. Specifically, it is achieved by running through the decision paths in tree *t*. On the root node in the path, *FC*_*t*,*k*_(**x**) = 0 and *f*_*t*_(**x**) is initialized to *β*_*t*_. Each time the instance arrives at a node with a decision branch on the *r*th feature, and *FC*_*t*,*r*_(**x**) will be incremented by the difference between the criterion scores at the child node along the path and the current node. Once the decision process of **x** reaches a leaf node, we assign a class to **x** and obtain all feature contributions along the decision path.

The prediction function of a forest, which is an ensemble of decision trees, takes the average of the predictions of its trees:

(7) $$\begin{array}{c} F\left(x\right)=\frac{1}{T}\overset{T}{\underset{t=1}{\sum }}f_{t}\left(x\right), \end{array}$$

where *T* is the number of trees in the forest. Similarly, the prediction function of a forest can also be decomposed with respect to feature contributions:

(8) $$\begin{array}{c} F\left(x\right)=\frac{1}{T}\overset{T}{\underset{t=1}{\sum }}\left(\overset{D}{\underset{k=1}{\sum }}{FC}_{t,k}\left(x\right)+\beta_{t}\right)=\overset{D}{\underset{k=1}{\sum }}\left(\frac{1}{T}\overset{T}{\underset{t=1}{\sum }}{FC}_{t,k}\left(x\right)\right)+\frac{1}{T}\overset{T}{\underset{t=1}{\sum }}\beta_{t}, \end{array}$$

where *FC*_*t*,*k*_ is the contribution of the *k*th feature in the *t*th tree. Therefore, the contribution of the *k*th feature to classify an instance **x** can be defined as

(9) $$\begin{array}{c} {\bar{FC}}_{k}\left(x\right)=\frac{1}{T}\overset{T}{\underset{t=1}{\sum }}{FC}_{t,k}\left(x\right), \end{array}$$

and the bias of the forest $\bar{\beta }=1/T\sum_{t=1}^{T}\beta_{t}$. The idea of interpreting the random forest model, which classifies sentences into *medication* or *symptom* category, is to find out those features with the most contribution to leading an instance to *medication* or *symptom* leaf nodes. We will not interpret *background* sentences since they are not as informative as the other two classes.

Suppose a random forest model *F*(**x**) is constructed given the training set *𝒳* = {(**x**_*i*_, *l*_*i*_) | 1 ≤ *i* ≤ *N*} with *N* labeled instances. To find out the important features for category *medication* and *symptom*, we select two subsets of training sets whose labels are *medication* and *symptom*, respectively. Let *𝒳*_*M*_ = {(**x**, *l*) | (**x**, *l*) ∈ *𝒳*, *l* = *medication*} be the subset of medication instances and *𝒳*_*S*_ = {(**x**, *l*) | (**x**, *l*) ∈ *𝒳*, *l* = *symptom*} be the subset of symptom instances; the average feature contributions for the two classes can be calculated as follows:

(10) $$\begin{array}{c} {\bar{FC}}_{M,k}=\frac{1}{\left|X_{M}\right|}\underset{\left(x,l\right)\in X_{M}}{\sum }{\bar{FC}}_{k}\left(x\right),\\ {\bar{FC}}_{S,k}=\frac{1}{\left|X_{S}\right|}\underset{\left(x,l\right)\in X_{S}}{\sum }{\bar{FC}}_{k}\left(x\right), \end{array}$$

where ${\bar{FC}}_{M,k}$ and ${\bar{FC}}_{S,k}$ are the positive contribution vectors of the *k*th feature for medication class and symptom class, respectively. After computing the contribution of features for each class, we rank these features to indicate their relative significance. Finally, the ones with larger contribution are selected as the discriminative features of each class.

### 4.3. Interpretation with Discriminative Patterns

To further exploit interpretability, we extract decision rules from the forest model to form a new space, where the forward selection is applied to select the top discriminative decision rule combinations, that is, discriminative patterns [8].

Specifically, a *pattern* is defined as the form of

(11) $$\begin{array}{c} \left(x_{i,j_{1}}\leq v_{j_{1}}\right)\wedge \left(x_{i,j_{2}}>v_{j_{2}}\right)\wedge \;\cdots \;\wedge \left(x_{i,j_{k}}\leq v_{j_{k}}\right), \end{array}$$

where *x*_*i*,*j*_ is the value on feature *j* of instance **x**_**i**_ and *v*_*j*_ is a scalar threshold. In our problem, a pattern can be any combination of rules from a decision tree. Furthermore, *discriminative patterns* (DPs) are those strong signaling patterns with high information gain or low Gini impurity in classification. In our problem, a pattern refers to a complete decision path, and a discriminative pattern is the path with low Gini impurity.

However, since the dimension |DP| of discriminative patterns is still high, we need to identify the most informative ones from them. To this end, we apply forward selection [19] to select the top *K* discriminative patterns. Let *f*_*s*_ be the forward selection function, then we have *f*_*s*_ : {0, 1}^|DP|^  →  {0, 1}^*K*^. We run *K* iterations, where the DP set at iteration *k* is denoted as Pat_*k*_. At iteration *I*, we traverse the discriminative patterns *dp*_*j*_ ∉ Pat_*I*−1_. A temporary DP set Pat_*Ij*_ at current iteration is built by adding *dp*_*j*_ to the DP set obtained in iteration *I* − 1, that is,

(12) $$\begin{array}{c} {Pat}_{Ij}={Pat}_{I-1}\;⋃\;\left\{dp_{j}\right\}. \end{array}$$

Then, we build a classifier using support vector machines [20] based on the selected patterns Pat_*Ij*_ and obtain the accuracy acc_*Ij*_ of the classifier. The best pattern *dp*_*j*∗_ is added into the DP set, where *j*^∗^ = arg max_*j*_Pat_*Ij*_ and acc_*Ij*∗_ > acc_*I*−1_, so that Pat_*I*_ = Pat_*Ij*∗_. After *K* iterations, we obtain the top *K* discriminative patterns Pat_*K*_. At last, each instance **x** in the dataset is mapped to the DP space as **y** ∈ {0, 1}^*K*^. If the *k*th pattern appears in **x**, then the corresponding entry **y**_*k*_ is set to 1; otherwise, 0.

## 5. Results and Discussions

In this section, first we present the experiments results which show that the forest-based models outperform the baseline methods. Second, we compare the interpretability between Lasso and our forest-based model by analyzing their discriminative features and discriminative patterns.

### 5.1. Experimental Setup

#### 5.1.1. Dataset

Since there are few datasets available for health-related texts classification, we created our dataset by collecting texts from online health communities to solve this problem. The data used for the experiment in this study were crawled from patient.info (http://patient.info/forums) using Scrapy, a python framework. The ground truth was obtained by assigning a label to each sentence in the data set. 257,187 discussions in 616 subforums from the forum were crawled. Then, we used NLTK tokenize package (http://www.nltk.org/api/nltk.tokenize.html) to split the texts in each discussion into a list of sentences. Given lists of sentences from all the discussions, we randomly select sentences from each list in portion and the number of selected sentences is 2585. We recruited two volunteers to complete the labeling work. Both volunteers were provided with the total 2585 randomly selected sentences and asked to categorize each of the sentences into medication, symptom, or others. The labeled sentences were merged based on unanimous voting. We discarded the sentences that were labeled with disagreements and obtained 2099 sentences categorized into the same label. The result of the sentences labeling is in Table 2. In the experiments, we set the label of class *background*, *medication*, and *symptom* to 0, 1, and 2, respectively.

#### 5.1.2. Baseline Methods

The contributions of our study we want to claim are how much improvement of the performance our proposed method can achieve by introducing the labeled sequential patterns as features and how the interpretability can be enabled by applying our proposed methods to sentence representatives in a variety of spaces to gain an insight of the health-related text classification model. To show the first contribution, we choose support vector machines trained on a variety of features proposed in [3]. We built binary classification SVM models for class *medication* and *symptom* with RBF kernel exp(−*γ*|*x* − *x*′|^2^), where *γ* is the reciprocal of the number of features. To predict an instance, the SVM models calculate the probabilities using Platt scaling. If the probabilities to classify the instance into *medication* and *symptom* are both less than 0.5, then we classify the instance into class *background*; otherwise, it is classified into the class with greater probability. In order to ensure the performance, we implement feature selection based on entropy using a decision tree model. In terms of the second contribution, we compare the model interpretability between Lasso [21] to random forests and DPClass and interpret the models using the features with nonzero weights in Lasso with L1 term coefficient set to 0.001.

#### 5.1.3. Evaluation Metrics

The metrics for the evaluation are accuracy, weighted average precision, weighted average recall, and weighted average *F*_1_ score. For multiclass classification, the weighted average precision, recall, and *F*_1_ score can be computed as follows:

(13) $$\begin{array}{c} precision=\frac{1}{N}\underset{l\in L}{\sum }N_{l}\;{precision}_{l,}\\ recall=\frac{1}{N}\underset{l\in L}{\sum }N_{l}\;{recall}_{l,}\\ F_{1}=\frac{1}{N}\underset{l\in L}{\sum }N_{l}F_{1l,} \end{array}$$

where *N* is the size of the test set, *L* is the label set, that is, *L* = {*medication*, *symptom*, *background*}, *N*_*l*_ is the size of the test subset with label *l*, and *precision*_*l*_, *recall*_*l*_, and *F*_1*l*_ are the precision, the recall, and the *F*_1_ score of the binary classification for instances with label *l*.

### 5.2. Classification Performance Evaluation

Table 3 shows the evaluation of each model using 5-fold cross validation. Each row represents the evaluation results of a model trained on data in different feature spaces. Each type of features used for training models are the ones selected with entropy-based methods, so that they are more informative and more discriminative in classification. For each model, the average accuracy (Acc), weighted average precision (Prec), recall (Rec), and F-score (F1) for medication class (M), symptom class (S), and the overall classes are presented, respectively.

For the SVM model, the entire average predicting accuracy achieves 79.8% with only word-based features, which outperforms the accuracies of Lasso. SVM also performs very well in terms of precision, recall, and F1 score. The model trained on LSP features alone fails to outperform the model trained on word-based features, but the former could achieve better performance than the latter if we add the UMLS semantic type features. Note that there are only hundreds of LSP features while there are more than 16 k word-based ones. Without feature selection, the performance of SVM is not very good, since the word-based features are considerably sparse. Furthermore, SVMs with RBF kernels do not provide interpretability directly for us to gain an insight of the sentences although the models achieve good performance.

From the experiment results using Lasso, we can find that the recall scores for classifying medication sentences are better than those for symptom ones, while the accuracies and precision scores indicate the opposite trend. As we use multiclass classifiers, many of the test instances are classified as medication class. The Lasso models trained on the word-based features slightly outperform the ones trained on the LSP features. As Table 4 shows, the weights of the LSP features are much smaller than those of the word-based ones in Lasso.

For the forest-based model, we can find that the accuracies of medication and symptom class can both achieve more than 80% with only LSP features and UMLS semantic type features. The overall accuracy achieves 80.9% and outperforms the other methods. Besides, with LSP and UMLS semantic type features, the precisions and recalls of both classes are greater than 0.8. Moreover, with position feature and word-based features, the performance of the forest-based model is even better. In general, the random forest model can achieve the relatively better F1 scores for both medication and symptom sentences classification. Similarly, the random forest models trained on the word-based features slightly outperform those trained on the LSP features.

Although it is not guaranteed that the models trained on LSP features outperform the ones trained on word-based features, we would still like to take advantage of LSP features since the feature dimension is significantly reduced without sacrificing the discrimination ability of models. In addition, LSP features provide a valuable perspective in both tag and structural levels to interpret classification results for health-related sentences.

### 5.3. Interpretability Evaluation

#### 5.3.1. Interpretability of Lasso

Table 4 lists the features with the largest weights in the combination of the word-based features, LSP features, UMLS Metathesaurus semantic type features, position feature, thread creator indicator feature, and word count features. After learning process, dedication-related features are assigned negative weights, while potential symptom-related features are assigned positive weights. Meanwhile, most of the word-based features have greater weights than the other features. The words “avoid,” “prescribe,” and “increase” are the most signaling words in medication sentences. The possible reason behind might be that medications usually require patients to avoid certain things, to take prescription drugs, or to adjust the dosages. The words such as “bleeding,” “anxiety,” “swelling,” “migraines,” and “fever” are common for symptom sentences in the forum, as they express external physical injury and mental diseases.

For LSPs, they are usually assigned with positive weights as they are capable of mining the symptom terms in the sentences. The pattern *(PRP*, *PRP*, *RB*, *SYMP)*, for example, is common for symptom-related sentences like “someone suffers from some symptom frequently/occasionally.” However, we also find that the tag *SYMP* is very frequent in both medication and symptom sentences, which is due to the reason that Lasso could not achieve good performance using LSP features, and is also hard to interpret the differences between class *medication* and class *symptom.*

Several UMLS semantic type features are assigned relatively larger weights to identify symptom sentences. For example, the term “sosy,” short for “sign or symptom,” is obviously a useful feature to identify symptom sentences. The term “mobd” (i.e., “mental or behavioral dysfunction”) can be used to detect mental disease symptoms. “patf” (i.e., “pathologic function”) is a parent semantic type of “mobd,” which is also an informative feature to detect pathologic terms.

#### 5.3.2. Interpretability of Forest-Based Model

To interpret healthcare-related sentences in forest-based models, we calculate the feature contributions from decision trees in the forest. We select one random forest model with the best accuracies in the experiments and list the 10 features with the greatest contributions for each class in Table 5.

In identifying *medication* sentences, the unigram feature “prescribed” has the largest contribution. This is because such kinds of sentences usually contain information about prescribing drugs. LSP features (*PRP*, *CD*, *CD*), (*PRP*, *CD*, *IN*, *NN*, *NN*), (*CD*, *IN*, *CD*, *CD*), and *(PRP*, *CD*, *JJ*, *JJ*) also contribute to recognizing sentences as medication-related ones as they all contain the POS tag *CD*, which represents the numbers in describing the dosages of medications. The morphological features are selected as the names of many drugs are capitalized terms or abbreviations. The UMLS semantic type feature “hlca” (i.e., “health care activity”) is important since healthcare activity terms are commonly seen in medication sentences. On the contrary, if a sentence does not contain LSP (*NN*, *SYMP*, *SYMP*, *CC*) or “sosy” (“sign or symptom”), or is not posted by the user (thr. Crt. = 0), this sentence may also be classified into medication class, as it is less likely to be symptom-related.

For the *symptom* class, the UMLS semantic type features “sosy” and “patf” are among the top relevant ones since they are capable of detecting symptom terms and pathologic terms, respectively. Thread creator indicator is also useful since symptom sentences are mainly posted by users to share their situations and ask for more information. If a sentence does not contain the word “prescribed,” then it is less likely to be medication-related. LSP features (*SYMP*, *SYMP*, *SYMP*), (*NN*, *SYMP*, *SYMP*, *CC*), and (*SYMP*, *CC*, *JJ*) are selected since there are usually multiple terms matching the tag *SYMP* in symptom sentences. The position feature is also important in identifying symptom class, as it is natural for users to mention their symptoms in the first *v*_th1_ posts, where *v*_th1_ is a threshold learned by the decision tree. Similarly, if the number of words from a sentence is greater than *v*_th2_ learned by the decision tree, the sentence will be more likely to be a symptom sentence.

Compared to the feature ranking in Lasso, we can have a better understanding from the feature contribution rankings for each class in the random forest. The relationships between features and classes can be learned from the feature contribution vectors while Lasso only provides the weights of the features, which may not be expressive enough to represent the relationships between features and classes. The random forest model can achieve both better performance and interpretability compared to Lasso.

DPClass [8] proposes to take further advantages of the discriminative patterns in a random forest built on the training set. The selected DPs can help users gain insights of the data. In the experiments, we chose *K* = 30 to obtain the top 30 DPs.  Table 6 lists the selected 10 DPs of a forest-based model trained on all proposed features. For example, considering the discriminative pattern ((RB, CD, IN, IN) = 0) ∩ ((VBP, IN, CD ,CD, NN) = 0) ∩ (“mg” = 0) ∩ (“prescribed” = 1) ∩ (dsyn = 0), if an instance satisfies each rule in the pattern, its corresponding DP feature entry will be set to 1. The existence of this pattern increase the likelihood of classifying the instance into medication class in the decision tree. From the patterns in the table, we can find that the terms matching tag *SYMP* are likely to occur in symptom sentences, while tags *CD* and *DRUG* often lead to nonsymptom leaves as they are more likely to occur in medication sentences. In another word, symptom sentences usually contain symptom terms while medication sentences usually contain drug terms and numbers which represent the dosages of the medications. In addition to LSP features, there are two conspicuous unigram patterns “anxiety” and “cough,” because the training set contains many sentences related to anxiety and cough conditions.

## 6. Related Work

Previous medication information extraction research mainly focused on extracting medication information from clinical notes, such as [22] using conditional random fields to identify named entities and support vector machines to build one-vs-one models [23], using a variety of drug lexicons and [24] using semantic tagging and parsing. Sondhi et al. [3] use conditional random fields and support vector machines to classify texts from online health forums. Wang et al. [25] propose an unsupervised method to extract adverse drug reactions on the online health forums. Bian et al. [26] propose to mine drug-related adverse events on large-scale tweets.

Our work focuses on both classifying and interpreting the online healthcare-related texts. The major challenges in healthcare-related text classification and interpretation are how to represent the texts and how to classify and interpret the data. For the former question, [3] proposes to use word-based features, semantic features, and other heuristic features. The problem of this representation is that word-based features have a huge dimension, but the data are usually sparse, which introduces considerable computation costs for feature selection and building models. Ding and Riloff [27] propose to represent the texts using word features, local context features, and web context features. In addition to the large and sparse data in word feature space, the web context features are generated online during the training process by querying Google and collecting titles and snippets, which could also introduce a significant amount of crawling and extracting computations and increase the feature representation dimension. A method to represent the texts in a space with low dimension is proposed in [10]. This method adopts the labeled sequential patterns as features and achieves both decent performance and efficiency. For the latter question, in terms of modeling and enabling the interpretability, Lasso [21] is proposed to enhance both the performance and the interpretability of regression models by tuning the parameter to shrink the features. Features with greater weights can be considered as more important, which enables the interpretability of the regression models. Tree-based and forest-based methods, for example, CART [17] and random forest [9], are also widely utilized to handle classifying and interpreting the data using the decision rules in the trees.

## 7. Conclusions and Future Work

In our research, we propose to use labeled sequential patterns to represent the healthcare-related sentences in order to reduce the dimension and sparsity of the data, which can both guarantee the performance and enhance the efficiency. Then, we build forest-based models on the training data which is capable of predicting with decent performance and interpreting the healthcare-related sentences by extracting the important features used in the decision rules, ranked by their contributions, and the discriminative patterns consist of the decision rules. Overall, the forest-based models trained on the proposed feature space can achieve good performance and enable the interpretability of the data. In the future, we will build a compact system based on this framework to help users directly extract and highlight the insightful sentences while they are viewing healthcare-related articles, posts, and so forth Moreover, we will also target to extract and interpret the insightful sentences from other categories such as medication effects and user questions and include data from other sources like clinical notes.

## Figures and Tables

**Algorithm 1:**
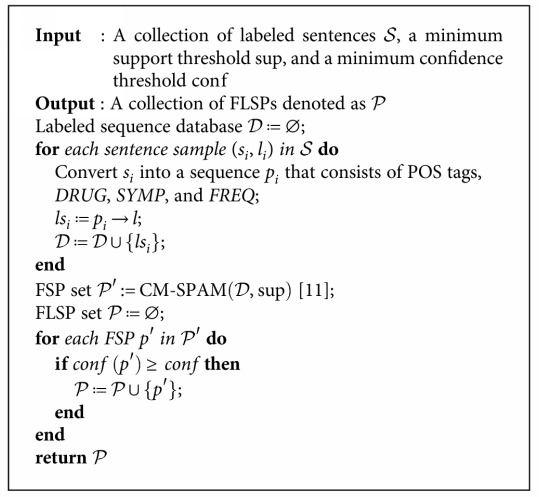
Frequent Labeled Sequential Patterns Generation.

**Table 1:** Tags introduction.

Tag	Description
*CC*, *CD*, *DT*, *EX*, and so on	Part-of-speech tags (https://www.ling.upenn.edu/courses/Fall_2003/ling001/penn_treebank_pos.html)
*DRUG*	Medications or drug terms (http://www.webmd.com/drugs/index-drugs.aspx?show=drugs)
*SYMP*	Symptom terms (http://symptomchecker.webmd.com/symptoms-a-z)
*FREQ*	Frequency phrases (customized regular expressions)

**Table 2:** Labeled sentences result.

Med.	Symp.	Others	Total
1127	772	200	2099

**Table 3:** Model evaluation. We evaluate each model using 5-fold cross validation. Each of the average accuracy, weighted average precision, weighted average recall, and weighted average F-score for medication class, symptom class, and the overall performance is presented in each column. Each row represents the performance of each model trained on different feature combinations.

	Ft. set	M. Acc.	M. Prec.	M. Rec.	M. F1.	S. Acc.	S. Prec.	S. Rec.	S. F1.	Acc.	Prec.	Rec.	F1.
Select + SVM	Word-based	0.843	0.846	0.867	0.856	0.886	0.875	0.804	0.838	0.798	0.808	0.798	0.802
	+ Semantic	**0.851**	0.854	**0.871**	**0.862**	0.884	0.874	0.801	0.836	0.804	0.816	0.804	0.808
	+ Position	0.843	0.846	0.867	0.856	0.886	0.875	0.805	0.838	0.798	0.808	0.798	0.802
	+ Thr. Crt.	0.844	0.846	0.867	0.857	0.896	**0.894**	0.814	0.852	0.800	0.812	0.800	0.805
	+ Morpho.	0.848	0.855	0.864	0.859	0.891	0.883	0.811	0.846	0.801	0.816	0.801	0.807
	+ Word Cnt.	0.802	0.785	**0.871**	0.826	0.864	0.888	0.722	0.796	0.761	0.773	0.761	0.763
	LSP	0.799	**0.894**	0.709	0.790	0.831	0.862	0.644	0.737	0.691	0.821	0.691	0.731
	+ Semantic	0.849	0.865	0.852	0.858	0.891	0.878	0.818	0.846	0.806	**0.823**	0.806	**0.813**
	+ Position	0.841	0.851	0.852	0.851	0.893	0.883	0.817	0.848	0.800	0.815	0.800	0.806
	+ Thr. Crt.	0.844	0.852	0.859	0.855	**0.897**	0.885	0.826	0.855	0.801	0.814	0.801	0.807
	+ Morpho.	**0.851**	0.860	0.864	0.861	0.896	0.883	0.826	0.854	**0.808**	0.820	**0.808**	**0.813**
	+ Word Cnt.	0.848	0.856	0.862	0.859	**0.897**	0.884	**0.830**	**0.856**	0.807	0.819	0.807	0.812
	+ Word-based	0.810	0.810	0.844	0.826	0.870	0.887	0.739	0.806	0.768	0.792	0.768	0.776

Lasso	Word-based	0.794	0.730	**0.979**	**0.837**	0.886	**0.969**	0.712	0.820	0.791	**0.785**	0.791	0.756
	+ Semantic	0.793	0.741	0.947	0.831	0.886	0.923	0.752	0.828	0.789	0.754	0.789	0.757
	+ Position	0.795	0.742	0.947	0.832	0.886	0.920	0.754	0.829	0.790	0.757	0.790	0.758
	+ Thr. Crt.	0.796	0.745	0.945	0.833	0.889	0.922	0.762	0.834	0.791	0.756	0.791	0.759
	+ Morpho.	0.797	0.745	0.947	0.834	0.889	0.924	0.759	0.833	0.792	0.757	0.792	0.760
	+ Word Cnt.	0.798	**0.746**	0.947	0.834	0.891	0.927	0.762	0.836	0.793	0.759	0.793	0.762
	LSP	0.715	0.663	0.955	0.782	0.802	0.875	0.538	0.666	0.711	0.678	0.711	0.665
	+ Semantic	0.769	0.712	0.955	0.816	0.861	0.911	0.689	0.785	0.767	0.727	0.767	0.728
	+ Position	0.767	0.710	0.955	0.814	0.860	0.910	0.686	0.782	0.765	0.716	0.765	0.725
	+ Thr. Crt.	0.771	0.715	0.953	0.817	0.864	0.911	0.700	0.791	0.769	0.728	0.769	0.731
	+ Morpho.	0.771	0.715	0.953	0.817	0.864	0.910	0.698	0.790	0.769	0.728	0.769	0.730
	+ Word Cnt.	0.771	0.715	0.953	0.817	0.864	0.910	0.698	0.790	0.769	0.728	0.769	0.730
	+ Word-based	**0.799**	0.745	0.950	0.835	**0.893**	0.930	**0.765**	**0.839**	**0.795**	0.759	**0.795**	**0.763**

Forest-based	Word-based	**0.848**	0.795	**0.969**	**0.873**	0.881	0.891	0.773	0.827	0.819	0.808	0.819	0.795
	+ Semantic	0.815	0.761	0.956	0.847	0.878	0.901	0.751	0.819	0.802	0.805	0.802	0.778
	+ Position	0.820	0.767	0.957	0.851	0.887	**0.908**	0.772	0.833	0.807	0.791	0.807	0.779
	+ Thr. Crt.	0.817	0.765	0.949	0.847	0.872	0.884	0.749	0.811	0.799	0.792	0.799	0.774
	+ Morpho.	0.832	0.776	0.965	0.860	0.890	0.907	0.781	0.838	0.816	**0.815**	0.816	0.789
	+ Word Cnt.	0.830	0.779	0.954	0.858	**0.893**	0.893	0.804	**0.846**	0.814	0.797	0.814	0.783
	LSP	0.786	0.742	0.921	0.822	0.863	0.861	0.748	0.801	0.771	0.725	0.771	0.739
	+ Semantic	0.837	0.824	0.887	0.854	0.879	0.860	0.802	0.829	0.809	0.805	0.809	**0.805**
	+ Position	0.840	**0.836**	0.873	0.854	0.882	0.844	**0.834**	0.839	0.808	0.800	0.808	0.803
	+ Thr. Crt.	0.832	0.825	0.875	0.849	0.879	0.849	0.814	0.831	0.802	0.796	0.802	0.797
	+ Morpho.	0.841	0.829	0.886	0.856	0.881	0.843	0.832	0.837	0.812	0.802	0.812	0.804
	+ Word Cnt.	0.829	0.816	0.881	0.847	0.880	0.856	0.808	0.831	0.800	0.791	0.800	0.793
	+ Word-based	0.848	0.816	0.927	0.868	0.887	0.861	0.827	0.843	**0.821**	0.803	**0.821**	0.802

**Table 4:** Top 10 average weight of word-based, LSP, semantic features in Lasso.

Word-based	Average weight	LSP	Average weight	Semantic	Average weight
Avoiding	−0.413	(PRP, PRP, RB, SYMP)	0.081	sosy	0.329
Wrong	−0.363	(PRP, PRP, VB, SYMP)	0.060	mobd	0.207
Avoid	−0.343	(VBZ, CC, SYMP)	0.058	patf	0.190
Prescribe	−0.323	(SYMP, SYMP, SYMP)	0.054	resa	−0.173
Bleeding	0.283	(PRP, SYMP, CC, SYMP, IN)	−0.053	inpo	0.100
Anxiety	0.281	(CC, SYMP, IN, SYMP)	−0.052	anab	0.094
Swelling	0.233	(PRP, SYMP, VBG)	0.049	mcha	−0.092
Increased	−0.185	(RB, SYMP, VB)	0.048	aggp	−0.090
Migraines	0.185	(JJ, IN, JJ, SYMP)	0.036	plnt	−0.063
Fever	0.160	(NN, SYMP, RB, SYMP)	−0.033	mamm	−0.052

**Table 5:** Top 10 feature contributions for medication and symptom class in a random forest model.

Feature	Back.	Med.	Sym.
*Top 10 FC for medication sentences*			
Prescribed = 1	−0.00275	0.01195	−0.00920
(PRP, CD, CD) = 1	−0.00251	0.01156	−0.00905
Morpho. = 1	−0.00206	0.00660	−0.00455
hlca = 1	−0.00071	0.00559	−0.00489
(NN, SYMP, SYMP, CC) = 0	0.00115	0.00429	−0.00544
sosy = 0	0.00191	0.00406	−0.00597
(PRP, CD, IN, NN, NN) = 1	−0.00075	0.00402	−0.00327
(CD, IN, CD, CD) = 1	−0.00120	0.00396	−0.00276
thr. Crt. = 0	0.00154	0.00381	−0.00535
(PRP, CD, JJ, JJ) = 1	−0.00086	0.00362	−0.00276
*Top 10 FC for symptom sentences*			
sosy = 1	−0.00589	−0.00783	0.01371
Prescribed = 0	0.00234	−0.015734	0.01339
thr. Crt. = 1	−0.00381	−0.00683	0.01064
(PRP, CD, CD) = 0	0.00271	−0.01264	0.00993
(SYMP, SYMP, SYMP) = 1	−0.00330	−0.00564	0.00895
(NN, SYMP, SYMP, CC) = 1	−0.00209	−0.00667	0.00876
Position < *v*_th1_	−0.00334	−0.00540	0.00874
patf = 1	−0.00254	−0.00379	0.00633
(SYMP, CC, JJ) = 1	−0.00172	−0.00404	0.00576
Word count > *v*_th2_	−0.00131	−0.00423	0.00554

**Table 6:** Top 10 discriminative patterns in a DPClass model.

Pattern	Leaf class
((RB, CD, CD) = 0) ∩ ((PRP, CD, CD, JJ) = 0) ∩ ((PRP, CD, NN, NN, NN) = 0) ∩ ((TO, VB, CD) = 1)	Med.
((IN, NN, NN, comma, SYMP) = 0) ∩ ((CD, RB, CD) = 1) ∩ ((RB, IN, IN, CD, IN) = 0) ∩ ((PRP, CC, CD, NN) = 1)	Med.
((SYMP, NN, VBG) = 1)	Sym.
((VBP, CD, NN, NN) = 0) ∩ ((SYMP, SYMP, NN) = 1)	Sym.
((RB, CD, IN, IN) = 0) ∩ ((VBP, IN, CD, CD, NN) = 0) ∩ (“mg” = 0) ∩ (“prescribed” = 1) ∩ (dsyn = 1)	Med.
((PRP, VBP, CD) = 0) ∩ ((CD, CD, NN, NN) = 1) ∩ ((TO, CD, IN) = 1)	Med.
(“cough” = 1)	Sym.
((RB, CD, IN, IN) = 0) ∩ ((VBP, IN, CD, CD, NN) = 0) ∩ (“mg” = 0) ∩ (“prescribed” = 0) ∩ (fndg = 0) ∩ ((NN, comma, comma, SYMP) = 1)	Sym.
((RB, CD, IN, IN) = 0) ∩ ((VBP, IN, CD, CD, NN) = 0) ∩ (“mg” = 0) ∩ (“prescribed” = 1) ∩ (dsyn = 0)	Med.
(“anxiety” = 1)	Sym.
